# Supplementary material for: A tissue-intrinsic IL-33/EGF circuit promotes epithelial regeneration after intestinal injury
Source: Nat Commun. 2023 Sep 5;14:5411. doi: 10.1038/s41467-023-40993-5 (PMC10480426; doi:10.1038/s41467-023-40993-5)
Supplement: Supplementary file 1 — Supplementary Information [file 41467_2023_40993_MOESM1_ESM.pdf]

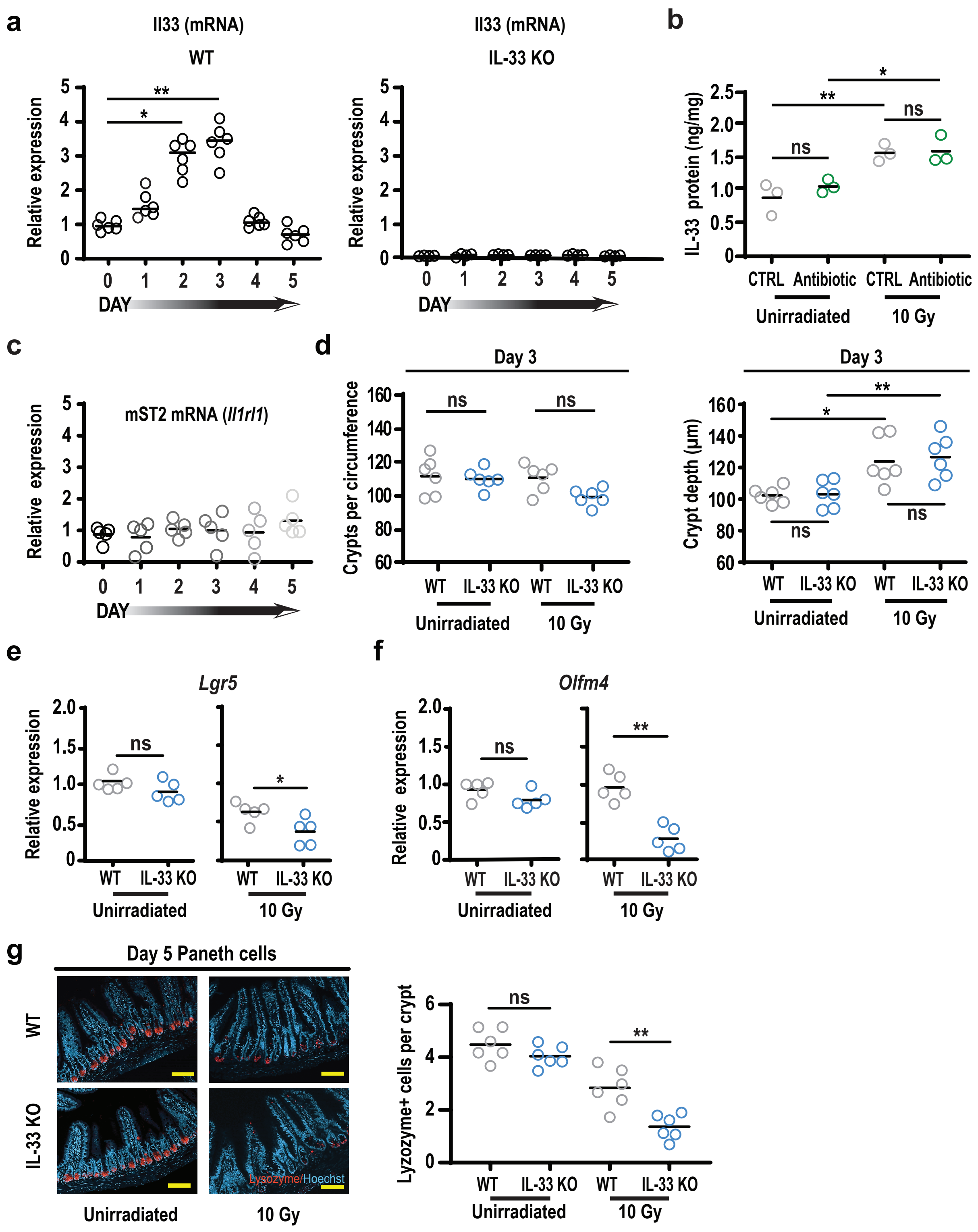

Figure S1. IL-33 quantification after radiation injury (10 Gy TBI). a, Time course of IL-33 expression in small intestine (SI) crypts isolated from WT and IL-33 KO mice after TBI; measured

by qPCR (n=6 WT and n=4 IL-33 KO mice/group). **b**, SI IL-33 protein from irradiated mice +/- pretreatment with Enrofloxacin and Ampicillin for one week prior to TBI (n=3 mice/group); measured by ELISA and normalized by the amount of total protein. **c**, Time course of membrane-bound ST2 expression in SI crypts isolated from WT mice after TBI; measured by qPCR (n=5 mice/group). **d**, Measurement of SI crypt number and depth in WT and IL-33 KO mice three days after TBI. Data combined from two independent experiments (n=6 mice/group). **e-f**, Relative expression of ISC markers *Lgr5* (**e**) and *Olfm4* (**f**) determined by qPCR in SI crypts isolated from WT and IL-33 KO mice at baseline or five days after irradiation (10 Gy); data combined from two experiments (n=5 mice per group). **g**, WT and IL-33 KO mouse ileum analyzed five days after TBI (10 Gy). Shown are quantification and representative immunofluorescent images of anti-lysozyme (red) and DAPI nuclear stain (blue) in WT and IL-33 KO mice before and after TBI (n=6 mice/group, combined from two independent experiments); scale bars: 100 µm. Statistical analyses performed using Kruskal-Wallis multiple comparison testing (**a, c**), ANOVA multiple comparison testing (**b, d, g**), or two-tailed Mann-Whitney U test (**e, f**). Graphs indicate the mean for each group; *p<.05, **p<.01, ***p<.001. Source data for graphs are provided in the Source Data file. The exact p values are as follows:

(**a**) comparisons made vs. day 0; WT day 0 vs day 2, p= 0.017; WT day 0 vs day 3, p= 0.034;
(**b**) unirradiated CTRL vs unirradiated Antibiotic, p=0.67; unirradiated CTRL vs 10 Gy CTRL, p=0.006; unirradiated Antibiotic vs 10 Gy Antibiotic, p= 0.03; 10 Gy CTRL vs 10 Gy Antibiotic, p=0.99;
(**d**) crypts per circumference, unirradiated WT vs unirradiated KO, p=0.99; crypts per circumference, 10 Gy WT vs 10 Gy KO, p=0.126; crypt depth, unirradiated WT vs unirradiated KO, p=0.99; crypt depth, unirradiated WT vs 10 Gy WT, p=0.02; crypt depth, unirradiated KO vs 10 Gy KO, p=0.009; crypt depth, 10 Gy WT vs 10 Gy KO, p=0.97;
(**e**) unirradiated, p=0.24; 10 Gy, p=0.047;
(**f**) unirradiated, p=0.15; 10 Gy, p=0.008;
(**g**) unirradiated WT vs unirradiated KO, p=0.547; 10 Gy WT vs 10 Gy KO, p=0.0012.

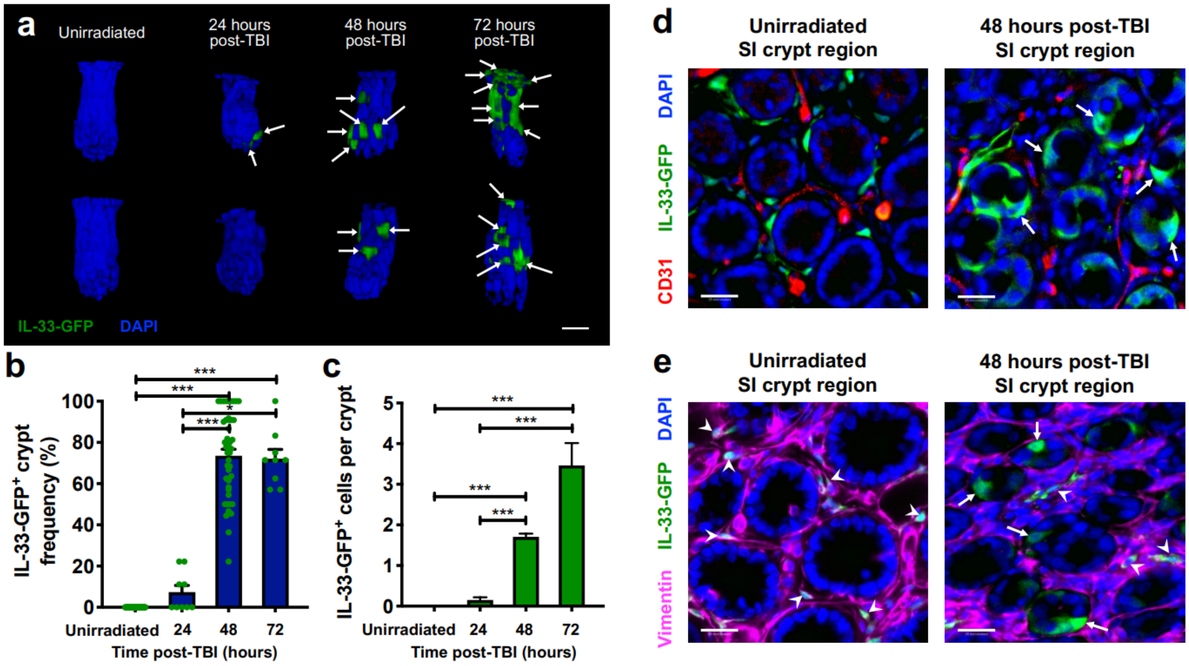

Figure S2. Ileal IL-33 expression within 72 hours of radiation injury. **a**, Representative three-dimensional (3-D) images of crypt IL-33-GFP expression during homeostasis and 24-72 hours after TBI (10 Gy); green: IL33-GFP$^+$ cells (anti-GFP staining); blue: nuclei (DAPI staining). **b**, Proportion of crypts with IL33-GFP$^+$ cells out of the total number of crypts per 3-D field; n=47 (Unirradiated), n=9 (24h), n=45 (48h), and n=9 (72h) independent 3-D fields. **c**, Quantification of IL33-GFP$^+$ cells within each crypt; n=379 (Unirradiated), n=73 (24h), n=476 (48h), and n=56 (72h) crypts/group. **d**, 2-D optical slices from 3-D imaging of crypt regions with staining for blood vessels (anti-CD31, red), indicating no overlap with IL-33-GFP expression (green); blue: nuclei (DAPI). **e**, 2-D optical slices from 3-D imaging of crypt regions with vimentin staining (magenta), indicating colocalization with stromal IL-33-GFP expression before and after irradiation, and no overlap with IL-33-GFP expression arising within crypts following irradiation; green: IL-33-GFP; blue: nuclei (DAPI). Arrowheads indicate IL33-GFP$^+$ fibroblasts (vimentin$^+$ cells). Arrows indicate IL33-GFP$^+$ crypt cells; scale bars: 25 μm. Statistical analyses performed using Kruskal-Wallis multiple comparison testing. Bar graphs show means, and error bars indicate SEM; *p<0.05 and ***p<0.001. Source data for graphs are provided in the Source Data file. The exact p values are:

(**b**) unirradiated vs 48h, p<0.0001; unirradiated vs 72h, p<0.0001; 24h vs 48h, p=0.0003; 24h vs 72h, p=0.0161;

(**c**) unirradiated vs 48h, $p<0.0001$; unirradiated vs 72h, $p<0.0001$; 24h vs 48h, $p <0.0001$; 24h vs 72h, $p<0.0001$.

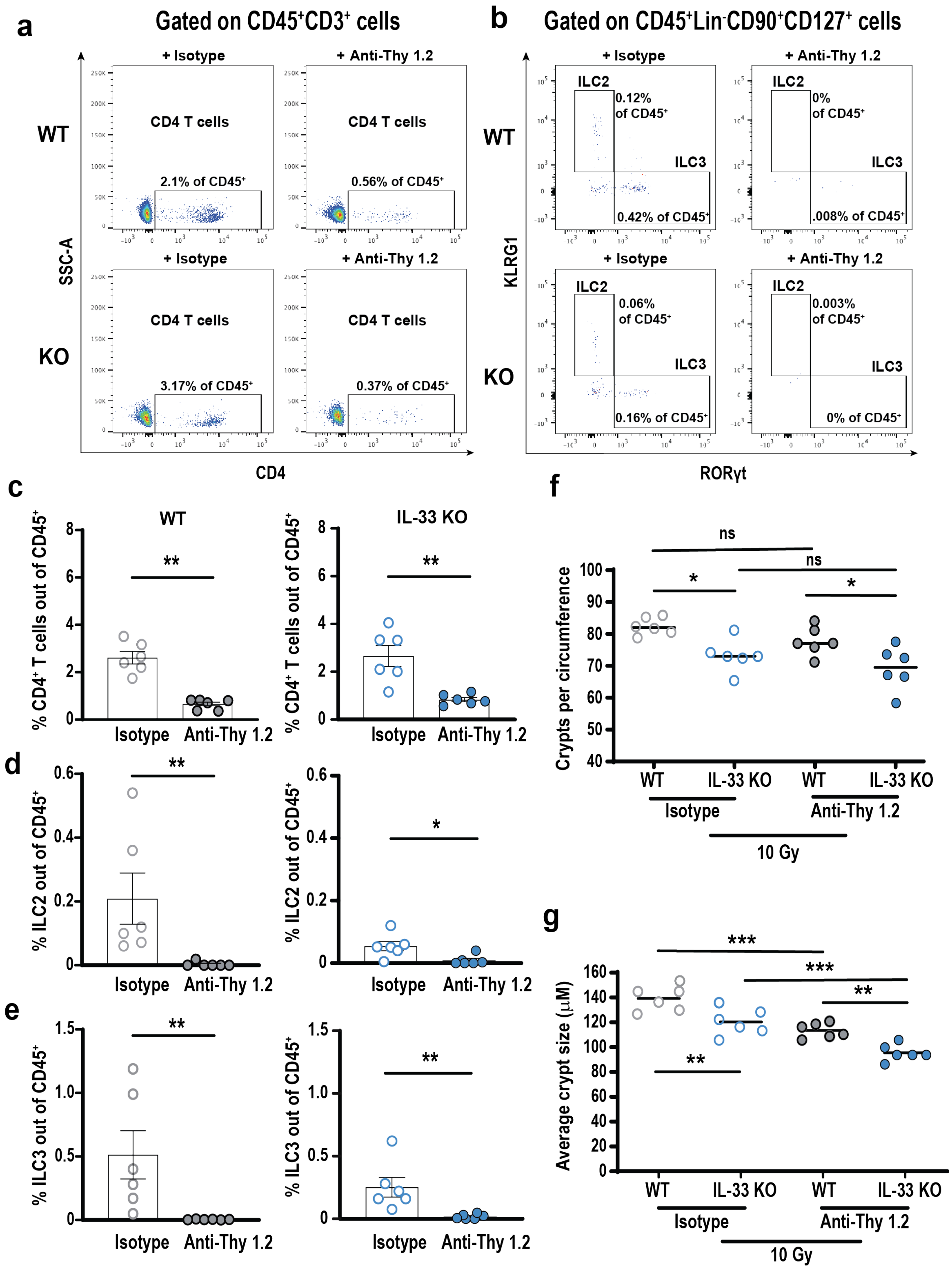

Figure S3. Anti-Thy1-mediated depletion of intestinal lymphocytes in WT and IL-33 KO mice. WT and IL-33 KO mice were treated with anti-Thy1.2 or isotype control antibodies on day

-4 and day -1 before TBI (10 Gy). Five days after irradiation, small intestines were harvested to perform flow cytometry and histologic analysis. **a**, Representative FACS plots of $CD4^+$ T cells (gated on live, $CD45^+CD3^+$ cells) isolated from intestinal lamina propria of WT and IL-33 KO mice treated with anti-Thy1.2 or isotype antibodies. **b**, Representative FACS plots of ILC2s (live, lineage-negative, $CD45^+CD90^+CD127^+KLRG1^+ROR\gamma t^-$ cells) and ILC3s (live, lineage-negative, $CD45^+CD90^+CD127^+ROR\gamma t^+KLRG1^-$ cells) isolated from intestinal lamina propria of WT and IL-33 KO mice treated with anti-Thy1.2 or isotype antibodies. Lineage-negative (Lin^-) cells were negative for CD3, CD19, CD11b, CD11c, Gr-1, and Ter-119 lineage-defining markers. **c,** $CD4^+$ T cell frequencies out of $CD45^+$ lamina propria lymphocytes (LPLs) isolated from WT or IL-33 KO mice. **d**, ILC2 frequencies out of $CD45^+$ LPLs isolated from WT or IL-33 KO mice. **e**, ILC3 frequencies out of $CD45^+$ LPLs isolated from WT or IL-33 KO mice. **f-g,** Average crypt number (**f**) and height (**g**) on day 5 after TBI (10 Gy) in mice pretreated with isotype or anti-Thy1.2 depleting antibodies. Statistical analyses were performed using two-tailed Mann-Whitney U test (**c-e**) or one-way ANOVA multiple comparison testing (**f, g**). Bar graphs indicate mean ±SEM; *p<.05, **p<.01, ***p<.001; n=6 mice/group combined from two independent experiments. Source data for graphs are provided in the Source Data file. The exact p values are:

(**c**) WT, p=0.002; KO, p=0.004;
(**d**) WT, p=0.002; KO, p=0.01;
(**e**) WT, p=0.002; KO, p=0.002;
(**f**) Isotype WT vs Isotype KO, p=0.017; Isotype WT vs Anti-Thy1.2 WT, p=0.3581; Isotype KO vs Anti-Thy1.2 KO, p=0.518; Anti-Thy1.2 WT vs anti-Thy1.2 KO, p=0.0317;
(**g**) Isotype WT vs Isotype KO, p=0.0056; Isotype WT vs Anti-Thy1.2 WT, p=0.0002; Isotype KO vs anti-Thy1.2 KO, p=0.0003; Anti-Thy1.2 WT vs anti-Thy1.2 KO, p=0.008.

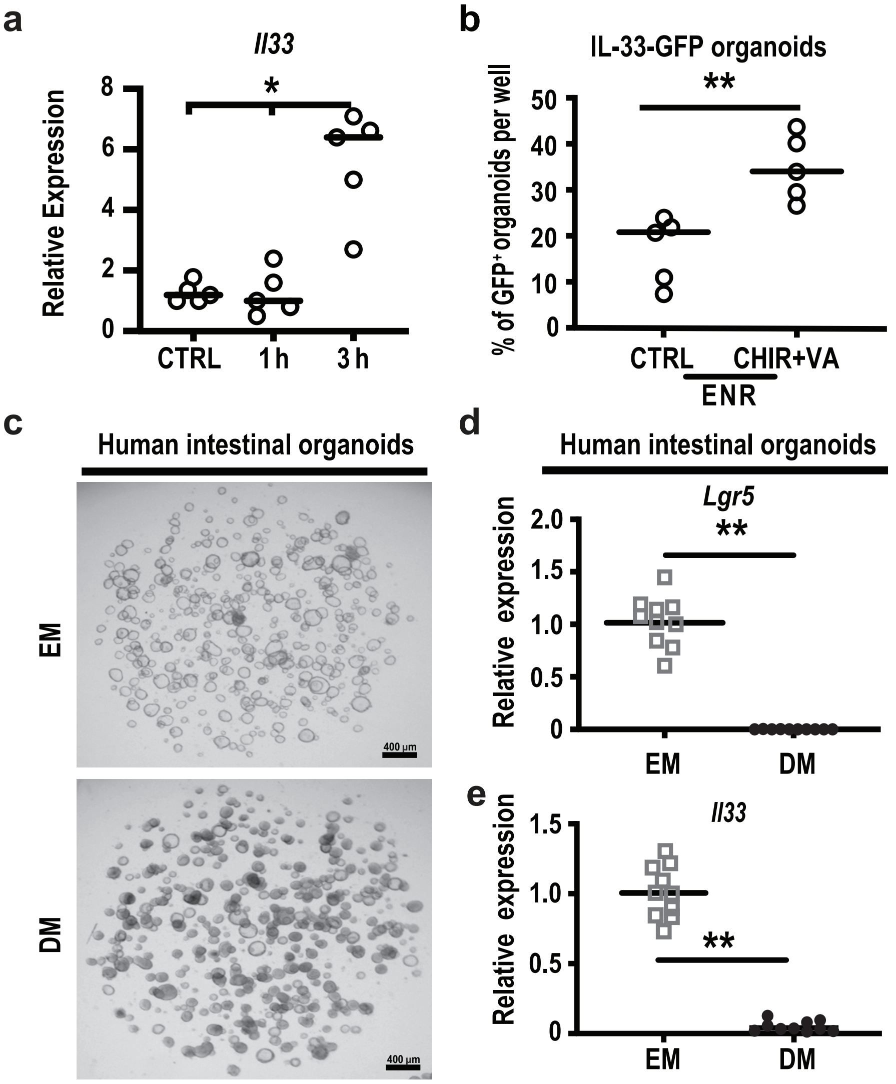

Figure S4. IL-33 expression *ex vivo*. a, Time course of IL-33 mRNA expression in WT mouse organoids after mechanical disruption (n=5 mice/group, combined from two experiments). **b**, Frequency of GFP-expressing organoids from IL-33-GFP mice after culture +/- CHIR99021 (CHIR, 3µM) and valproic acid (VA, 1mM) for 48 hours; data combined from two experiments (n=5 wells/group). **c**, Representative images of human ileal organoids cultured in expansion media (EM), which promotes ISC expansion, or differentiation media (DM), which promotes differentiation into mature enterocytes. **d-e**, Relative expression of *Lgr5* (**d**) and *Il33* (**e**) in human ileal organoids cultured in EM or DM (n=10 wells/group). Graphs show individual values and means. Statistical analyses were performed using Kruskal-Wallis multiple comparison testing (**a**),

unpaired t-test (**b**), or two-tailed Wilcoxon test (**d, e**); *p<.05, **p<.01. Source data for graphs are provided in the Source Data file. The exact p values are:

(**a**) CTRL vs 3h, p=0.039; 1h vs 3h, p=0.014;
(**b**) CTRL vs CHIR+VA, p=0.005;
(**d**) EM vs DM, p=0.002;
(**e**) EM vs DM, p=0.002.

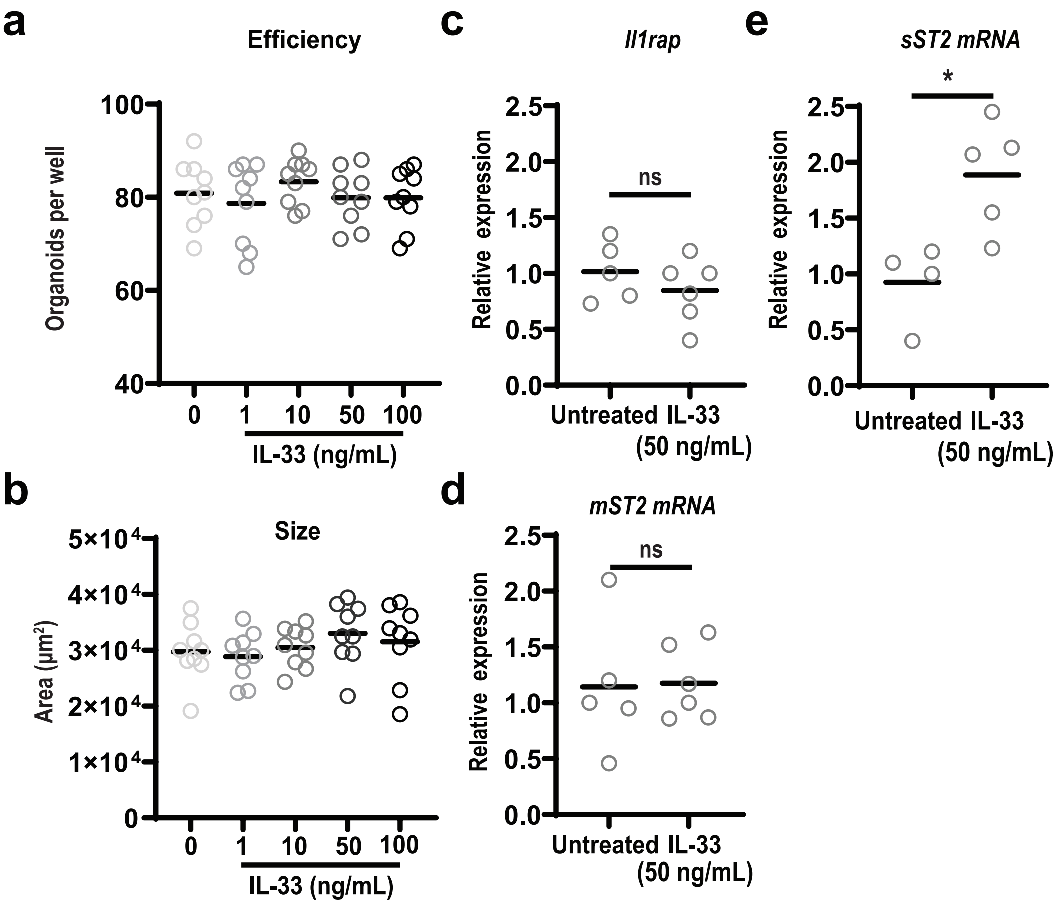

Figure S5. Mouse small intestine organoid cultures +/- IL-33. a-b, WT organoid number (**a**) and size (**b**) (n=9 wells/group) were assessed after five days in culture with ENR +/- IL-33; data combined from three experiments (n=9 wells/group). **c-e**, WT organoids assayed by qPCR after culture in ENR +/- IL-33; showing expression of the membrane-bound IL-33 receptor subunits *Il1rap* (**c**) and mST2 (**d**), combined from two experiments (n=5 untreated and n=6 IL-33-treated wells/group), and expression of sST2 (**e**), combined from two experiments (n=4 untreated and n=5 IL-33-treated wells/group). Statistical analyses were performed using one-way ANOVA multiple comparison testing (**a, b**) or two-tailed Mann-Whitney U test (**c-e**). Plots show individual values and means for each group; *p<.05. Source data for graphs are provided in the Source Data file. The exact p values are: p=0.4502 (**c**), p=0.9697 (**d**), and p=0.016 (**e**).

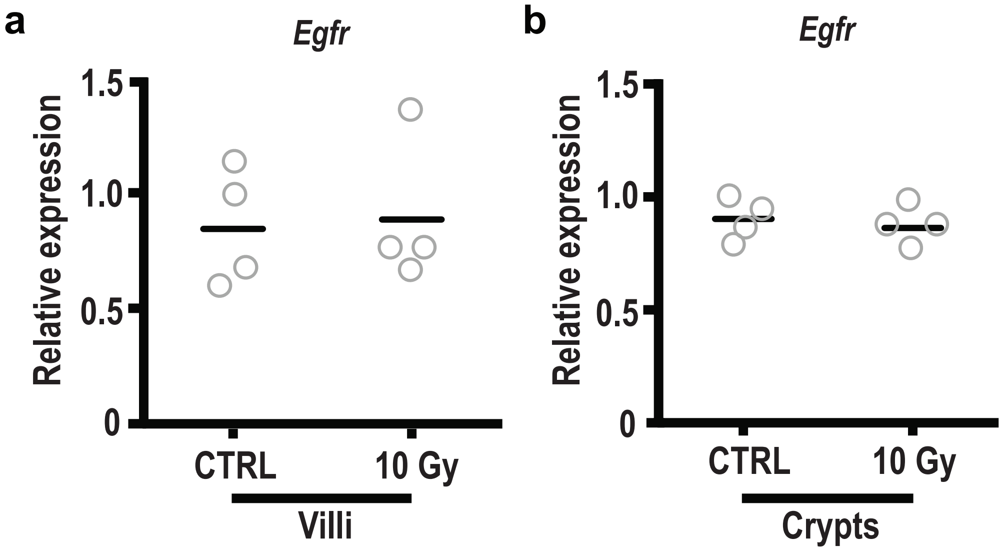

Figure S6. Radiation injury does not change intestinal expression of *Egfr*. qPCR for *Egfr* in villi (**a**) and crypts (**b**) isolated from small intestine five days after TBI (10 Gy); data combined from two experiments (n=4 mice/group). Comparisons were performed with the two-tailed Mann-Whitney U test. Source data for graphs are provided in the Source Data file. Graphs indicate the mean for each group.

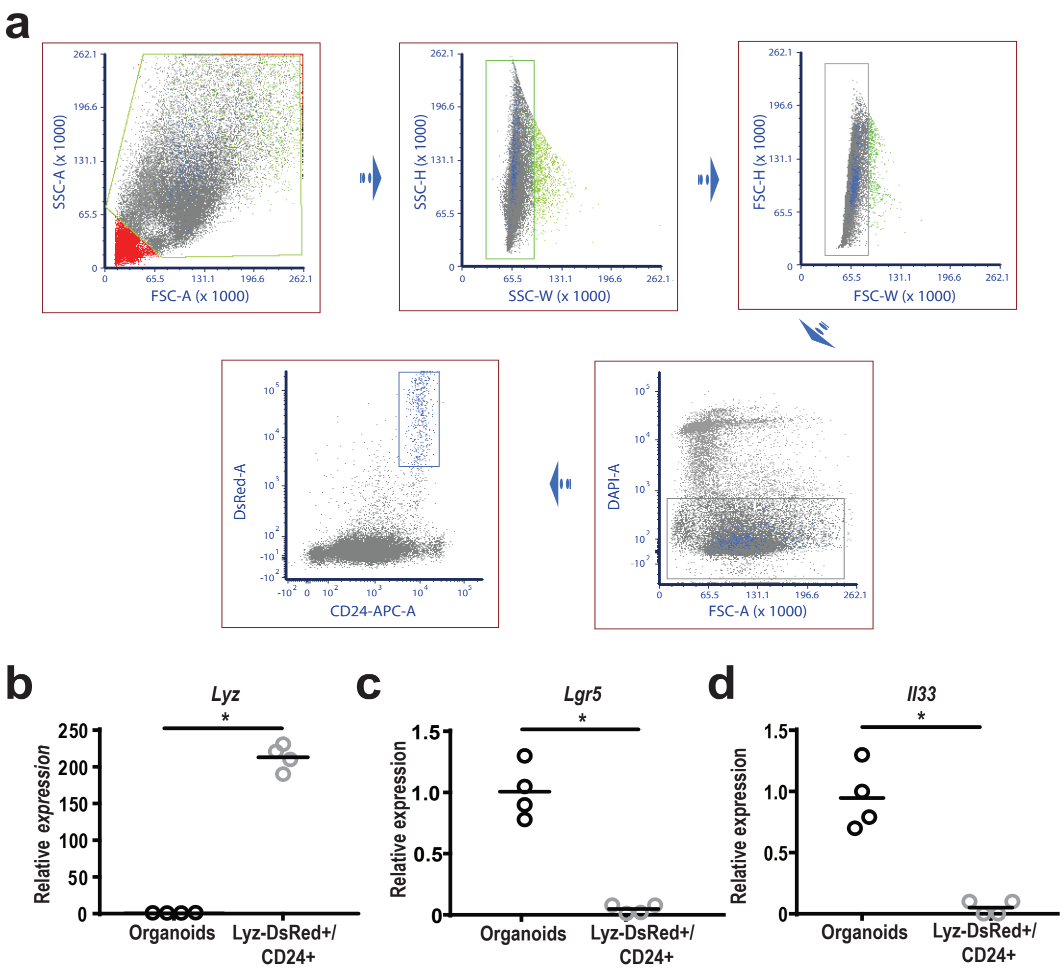

Figure S7. Gene expression in sorted Paneth cells. a, Paneth cell gating strategy: isolation of Lysozyme$^+$CD24$^+$ Paneth cells from Lyz-DsRed mice by FACS after staining with anti-CD24 (APC). **b-d**, qPCR of sorted Paneth cells and intact organoid controls, showing relative expression of *Lyz1/2* (**b**), *Lgr5* (**c**) and *Il33* (**d**); data combined from two experiments (n=4 mice/group). Comparisons were performed using the two-tailed Mann-Whitney U test. Graphs indicate the mean for each group; *p<.05. Source data for graphs are provided in the Source Data file. The exact p values are:

(**b**) Organoids vs Lyz-DsRed$^+$, p=0.0286;
(**c**) Organoids vs Lyz-DsRed$^+$, p=0.0286;
(**d**) Organoids vs Lyz-DsRed$^+$, p=0.0286.

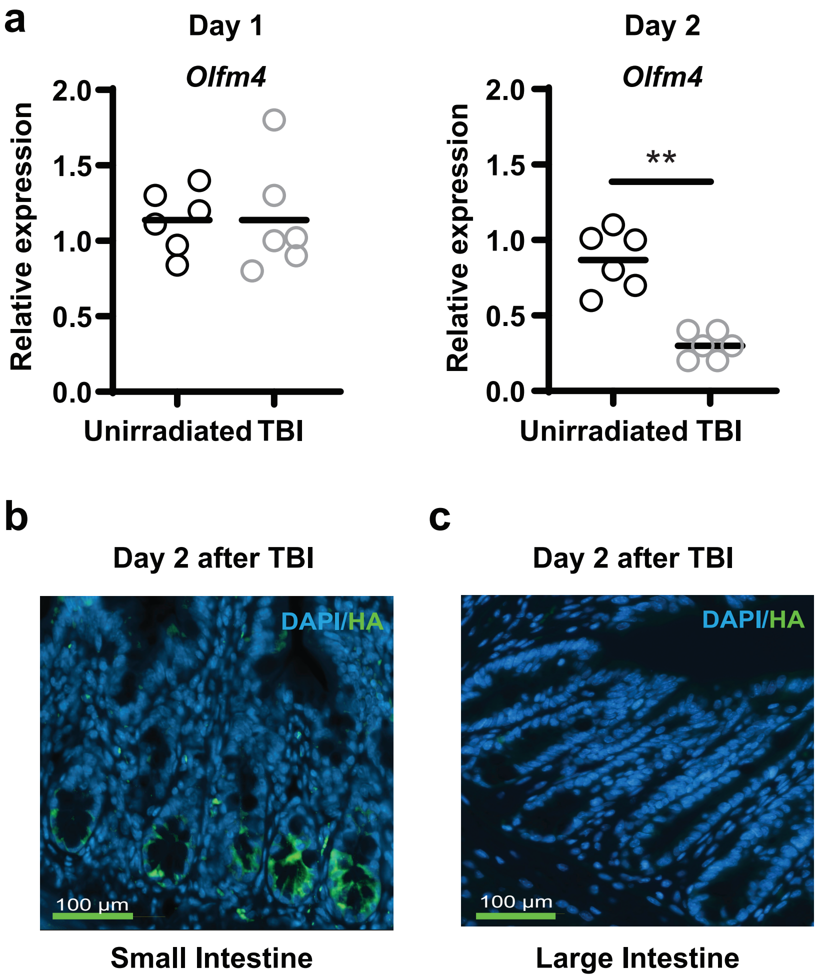

Figure S8. Stem cell persistence after radiation injury. a, Time course of *Olfm4* expression in small intestine crypts isolated from WT mice after TBI; measured by qPCR (n=6 mice/group). **b**, Representative image from Olfm4-Ribo small intestine (ileum) 48 hours after TBI (n=3 mice/group); blue: nuclei (DAPI), green: anti-hemagglutinin (HA). **c**, Representative image from Olfm4-Ribo large intestine 48 hours after TBI (n=3 mice/group); blue: nuclei (DAPI), green: anti-hemagglutinin (HA). Comparisons were performed with two-tailed Mann-Whitney U test. Plots show individual values and the means; **p<0.01. Source data for graphs are provided in the Source Data file. The exact p values are as follows:

(**a**) unirradiated vs Day 1 post-TBI, p=0.73;
(**b**) unirradiated vs Day 2 post-TBI, p=0.002.

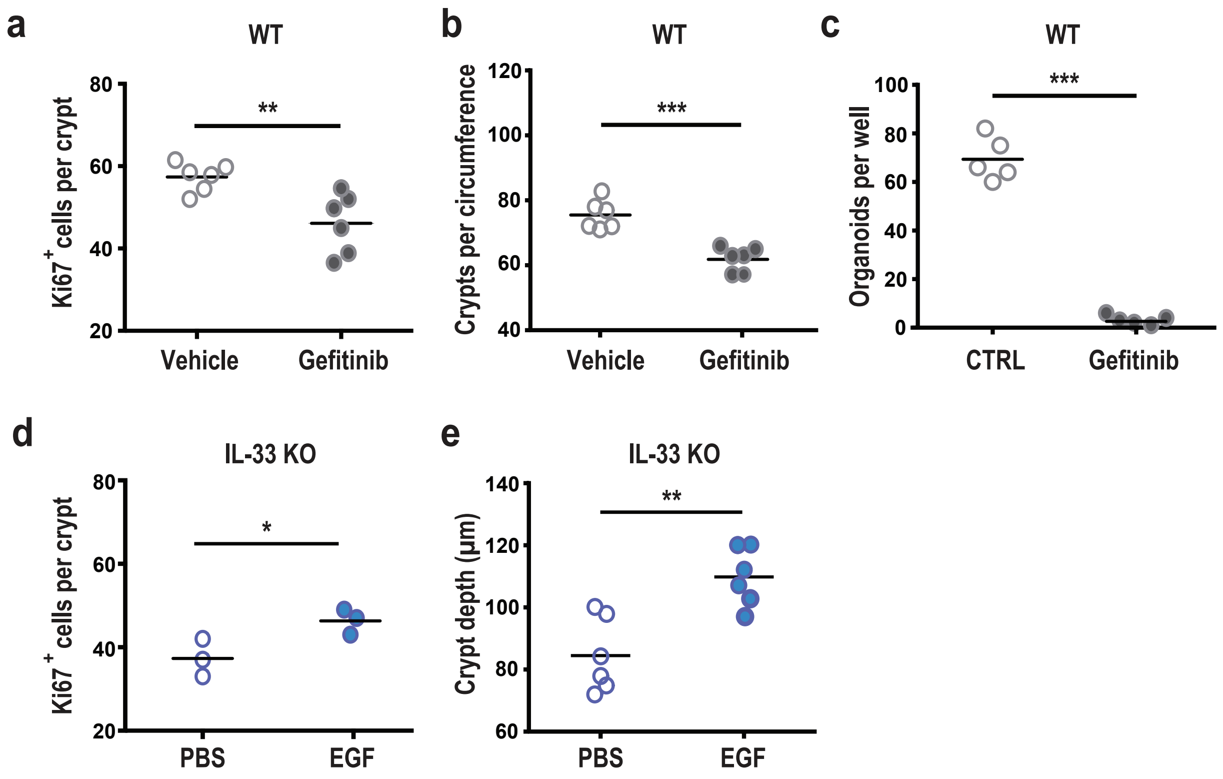

Figure S9. EGF and IL-33 in epithelial regeneration. a-b, Ileum from irradiated WT mice (10 Gy TBI) +/- gefitinib treatment (1 mg/mouse) or vehicle (daily for three days starting the day of irradiation). **a**, Average number of $Ki67^+$ cells per crypt on day five after irradiation of WT mice; combined from two independent experiments (n=6 mice/group). **b**, Crypt quantification in WT mice on day five following irradiation; data combined from two experiments (n=6 mice/group). **c**, Total number of organoids per well generated from single cells after culture for five days +/- gefitinib (1 µM); data combined from two experiments (n=5 wells/group); "CTRL" group represents gefitinib-negative control cultures. **d-e**, Ileum from irradiated IL-33 KO mice (10 Gy TBI) treated with EGF (10 µg/mouse) or PBS (daily for three days starting the day of irradiation). **d**, Average number of $Ki67^+$ cells per crypt five days after irradiation of IL-33 KO mice and daily treatment for three days (from day 0-2) with EGF or PBS (n=3 mice/group). **e**, Crypt depth in irradiated IL-33 KO mice treated with EGF or PBS daily for three days (from day 0-2); data combined from two independent experiments (n=6 mice/group). Graphs indicate the mean for each

group; comparisons performed by two-tailed unpaired t-test; *p<.05, **p<.01, ***p<.001. Source data for graphs are provided in the Source Data file. The exact p values are:

(**a**) Vehicle vs Gefitinib, p=0.0068;
(**b**) Vehicle vs Gefinitib, p=0.0003;
(**c**) CTRL vs Gefinitib. p<0.0001;
(**d**) PBS vs EGF. p=0.048;
(**e**) PBS vs EGF. p=0.0021.

Table S1. Antibodies utilized for flow cytometry.

Antigen	Fluorochrome	Clone	Company	Catalog #	Dilution
CD11b	Biotin	M1/70	BD Biosciences	557395	1:1600
CD11c	Biotin	HL3	BD Biosciences	553800	1:200
CD127	APC-eFluor 780	A7R34	eBioscience	47-1271-82	1:50
CD19	Biotin	1D3	BD Biosciences	553784	1:800
CD3	PE-eFluor 610	145-2C11	eBioecience	61-0031-80	1:100
CD4	Brilliant Violet 711 (BV711)	RM4-5	BioLegend	100549	1:400
CD45	Brilliant Violet 785 (BV785)	30-F11	Biolegend	103149	1:400
CD90.2	FITC	53-2.1	Biolegend	140303	1:200
Fixable Viability Dye	eFluor 455 UV		eBioscience	65-0868-14	1:500
KLRG1	Brilliant Violet 605 (BV605)	2F1/KLRG1	Biolegend	138419	1:50
Ly-6G and Ly-6C (Gr-1)	Biotin	RB6-8C5	BD Biosciences	553124	1:1600
ROR gamma (t)	APC	AFKJS-9	eBioscience	17-6988-82	1:100
Steptavidin Conjugate	Qdot 655		Invitrogen	Q10123MP	1:800
Ter-119	Biotin	TER-119	BD Biosciences	553672	1:400
CD24	APC	M1/69	BioLegend	101813	1:200
